# Anaphylactic Death: A New Forensic Workflow for Diagnosis

**DOI:** 10.3390/healthcare9020117

**Published:** 2021-01-22

**Authors:** Massimiliano Esposito, Angelo Montana, Aldo Liberto, Veronica Filetti, Nunzio Di Nunno, Francesco Amico, Monica Salerno, Carla Loreto, Francesco Sessa

**Affiliations:** 1 Legal Medicine, Department of Medical, Surgical and Advanced Technologies, “G.F. Ingrassia”, University of Catania, 95123 Catania, Italy; massimiliano.esposito91@gmail.com (M.E.); angelomontana49@gmail.com (A.M.); aldoliberto@gmail.com (A.L.); francescoamico08@gmail.com (F.A.); 2 Human Anatomy and Histology, Department of Biomedical and Biotechnology Sciences, University of Catania, 95123 Catania, Italy; verofiletti@gmail.com (V.F.); carla.loreto@unict.it (C.L.); 3 Department of History, Society and Studies on Humanity, University of Salento, 73100 Lecce, Italy; nunzio.dinunno@icloud.com; 4 Department of Clinical and Experimental Medicine, University of Foggia, 71122 Foggia, Italy; francesco.sessa@unifg.it

**Keywords:** anaphylactic death, diagnostic workflow, immunohistochemical investigation, blood tryptase level

## Abstract

Anaphylaxis is a life-threatening or fatal clinical emergency characterized by rapid onset, and death may be sudden. The margin of certainty about the diagnosis of anaphylactic death is not well established. The application of immunohistochemical techniques combined with the evaluation of blood tryptase concentrations opened up a new field of investigation into anaphylactic death. The present study investigated eleven autopsy cases of anaphylactic death, carried out between 2005 and 2017, by the Departments of Forensic Pathology of the Universities of Foggia and Catania (Italy). An analysis of the medical records was carried out in all autopsies. Seven autopsies were carried out on males and four on females. Of the eleven cases, one showed a history of asthma, one of food ingestion, two of oral administration of medications, six did not refer any allergy history, and one subject was unknown. All cases (100%) showed pulmonary congestion and edema; 7/11 (64%) of the cases had pharyngeal/laryngeal edema and mucus plugging in the airway; only one case (9%) had a skin reaction that was found during external examination. Serum tryptase concentration was measured in ten cases, and the mean value was 133.5 µg/L ± 177.9. The immunohistochemical examination using an anti-tryptase antibody on samples from the lungs, pharynx/larynx, and skin site of medication injection showed that all cases (100%) were strongly immunopositive for anti-tryptase antibody staining on lung samples; three cases (30%) were strongly immunopositive for anti-tryptase antibody staining on pharyngeal/laryngeal samples; and eight cases (80%) were strongly immunopositive for anti-tryptase antibody staining on skin samples. We conclude that a typical clinical history, blood tryptase level >40 µg/L, and strongly positive anti-tryptase antibody staining in the immunohistochemical investigation may represent reliable parameters in the determination of anaphylactic death with the accuracy needed for forensic purposes.

## 1. Introduction

The term anaphylaxis was introduced in 1902 by Portier and Richet [1], and it refers to a serious, generalized or systemic, allergic or hypersensitivity reaction [2]. It can be a life-threatening or fatal clinical emergency with airway and circulatory impairments [3,4,5,6]. It is usually associated with skin and mucosal alterations (widespread hives, pruritus, and swollen lips/tongue/uvula) and gastrointestinal disorders (vomiting, diarrhea, and abdominal cramps) [7,8]. In particular, anaphylaxis is due to a systemic reaction mediated by vasoactive amines, released from mast cells, and basophils sensitized by immunoglobulin E (IgE) [7,9,10,11,12]. Conversely, anaphylactic shock (AS) is an anaphylactic reaction characterized by critical organ hypoperfusion after exposure to a previously encountered antigen [11,12]. The incidence and prevalence of anaphylaxis are difficult to establish. However, the incidence ranges from 1.5 to 7.9 per 100,000 person-years, but there has been an increase in admissions with anaphylaxis over the last two decades [3]. Moreover, the prevalence is 0.3% in the European population [3]. According to Chaudhuri et al. [13], the incidence of anaphylaxis in the United States ranged from 1.21% to 15.04% in the population. Furthermore, the risk of severe anaphylaxis has been estimated to be 1–3 per 10,000 person-years, while the risk of death due to anaphylactic shock is about 1–3 per million per year. Food and medications are responsible for most anaphylaxis reactions. However, virtually any agent capable of directly or indirectly activating mast cells or basophils can cause this syndrome [9,10,11,12,13,14,15,16,17,18]. Food is the cause of anaphylaxis in children most of the time, and drugs are major causes in adults, and are also the most frequent cause of anaphylaxis in hospitalized patients [12,19,20,21,22]. A higher frequency of anaphylaxis has been shown in adult females to food and non-steroidal anti-inflammatory drugs (NSAIDs) [8,12]. The common drugs responsible for anaphylaxis reactions are antibiotics, muscle relaxants, non-steroidal anti-inflammatory drugs, and radioactive contrast media [8,13]. Risk factors for severe anaphylaxis with hospitalization are old age combined with comorbidities such as cardiovascular disease (CVD) and chronic obstructive pulmonary disease [14,15,22,23,24,25,26].

Tryptase is an abundant secretory granule-derived serine proteinase contained in mast cells. The tryptase enzyme is the only protein that is specific for human mast cells, and tryptase plasma levels reflect the clinical severity of anaphylaxis. Elevated levels of serum tryptase occur in both anaphylaxis and anaphylactic reactions, but a negative test does not exclude anaphylaxis [27].

In this study, an investigation of the serum tryptase levels combined with the immunohistochemical expression of tryptase in specimens from the lungs, glottis, and skin (site of medication injection) in eleven autopsy cases was performed to clarify and discuss their significance in anaphylactic death.

## 2. Materials and Methods

### 2.1. Sample Collection

A retrospective analysis of the autopsy records of the Departments of Forensic Pathology of the University of Foggia and Catania (Italy), was carried out between 2005 and 2017. From the analysis of death scene investigations and autopsy reports, together with the information gathered from the police, eleven cases of anaphylactic death origin were selected. Cases with weak or missing information about the manner of death were excluded. Decomposed bodies were also excluded from the study. All procedures performed in this study were in accordance with the ethical standards of the institution and with the 1964 Helsinki Declaration and its later amendments or comparable ethical standards. Informed consent was obtained from all relatives.

The deceased were four men and seven women, ranging in age from 16 to 69 years (average: 47 years ± 17.69). In all cases of anaphylactic death, sections of lungs, glottis and the skin site of medication injection were collected. Eleven cases were selected as controls. These included the following cases: seven cases of sudden cardiac death and four cases of fatal motor vehicle crashes. All control cases were selected for their negative clinical histories for manifestations of asthma or allergies. All autopsies were performed within four days after death was determined, and all cadavers were stored at −4 °C.

### 2.2. Histological Analysis

A routine microscopic histopathological study was performed using hematoxylin–eosin (H&E) staining. Specimens from the lungs, glottis and skin were fixed in 10% buffered formalin, as previously described [28]. After an overnight wash, specimens were dehydrated in graded ethanol, cleared in xylene and paraffin-embedded. Tissue paraffin blocks were then cut (4 μm thickness) using a microtome and sections were mounted on silane-coated slides (Dako, Glostrup, Denmark) and stored at room temperature. Sections then were stained with H&E and observed using a Zeiss Axioplan light microscope (Carl Zeiss, Oberkochen, Germany) for morphological examination. Finally, representative micrographs were captured using a Zeiss AxioCam MRc5 digital camera (Carl Zeiss, Oberkochen, Germany).

### 2.3. Immunohistochemical Staining

Immunohistochemical investigation of samples from the lungs, glottis and skin was performed using anti-tryptase antibodies. For the immunohistochemical analysis, specimens were processed as previously described [28,29]. In particular, sections were dewaxed in xylene, rehydrated with graded ethanol and then incubated for 20 min in 0.3% H_2_O_2_/methanol solution to block endogenous peroxidase activity. After rinsing for 20 min with phosphate buffered saline (PBS), slides were pre-treated to facilitate antigen retrieval and to increase membrane permeability to antibodies using a microwave oven (750 W) (5 min × 3) in capped polypropylene slide-holders with citrate buffer (10 mM citric acid, 0.05% Tween 20, pH 6.0; Bio-Optica, Milan, Italy) and then incubated overnight at 4 °C with anti-tryptase monoclonal antibodies (Agilent Dako, Copenhagen, Denmark) diluted 1:100 in PBS. The detection system used was the LSAB+ kit (Dako, Copenhagen, Denmark) incubated for 10 min at room temperature, a refined avidin–biotin technique in which a biotinylated secondary antibody reacts with several peroxidase-conjugated streptavidin molecules. The positive reaction was visualized by 3,3-diaminobenzidine (DAB) peroxidation (DAB substrate Chromogen System; Dako) according to standard methods [30]. The sections were counterstained with Mayer’s hematoxylin (Histolab Products AB, Göteborg, Sweden) mounted in GVA (Zymed Laboratories, San Francisco, CA, USA). The sections were observed and photographed as described above.

### 2.4. Evaluation of Immunohistochemistry (IHC)

The anti-tryptase immunoreaction was identified as either negative or positive. Immunohistochemical positive staining was defined by the presence of brown chromogen detected on the edge of the hematoxylin-stained cell nucleus, distributed within the cytoplasm or in the membrane via evaluation by light microscopy. Positive controls consisted of tissue specimens with known antigenic positivity. Sections treated with PBS without any primary antibody served as negative controls. Seven fields of about 600,000 µm^2^, randomly selected from each section, were considered for morphometric and densitometric analysis. The percentage of the areas (morphometric analysis) stained with anti-tryptase antibody was expressed as % positive dark brown pixels of the analyzed fields. Moreover, the levels (high/low) of staining intensity of positive areas (densitometric analysis) were expressed as densitometric count (pixel^2^) of positive dark brown pixels in the analyzed fields. These parameters were calculated using software for image acquisition (AxioVision Release 4.8.2-SP2 Software, Carl Zeiss Microscopy GmbH, Jena, Germany). Data are expressed as mean ± standard deviation (SD). Digital micrographs were taken and fitted as previously described. The samples were also examined with a confocal microscope, and a three-dimensional reconstruction was performed (True Confocal Scanner, Leica TCS SPE).

### 2.5. Statistical Analysis

Statistical analysis was performed using GraphPad Prism 7.0 (GraphPad Software, Inc., La Jolla, CA, USA). The Shapiro–Wilk normality test was used for the calculation of the distribution of the samples. Unpaired t-tests were used for the comparison between the levels of tryptase staining intensity of positive areas (pixel^2^) of cases of anaphylactic death and controls. *p*-values of less than 0.05 (*p* < 0.05) were considered significantly different.

### 2.6. Serum Tryptase Assay

Samples of femoral blood were obtained via a transcutaneous femoral approach (from the femoral artery) in eleven post-mortem examinations, of which all subsequently underwent full autopsy. Serum was derived from whole blood by centrifugation, decanted into plastic test-tubes and stored at −80 °C. Samples were shipped on ice to the Industrial Bio-Test (IBT) Reference Laboratory (Florence, Italy) for analysis. Information regarding the cause of death was hidden from the reference laboratory performing the assays. Serum tryptase levels were determined using a competitive immunofluorescent enzyme assay with monoclonal anti-human tryptase antibodies against both the A and B structural types of tryptase. These antibodies were incubated with a serum aliquot; the sample was washed, and enzyme-labelled anti-tryptase was added, followed by incubation. The sample was washed a second time, the developer was added, and the fluorescence in the aliquot was measured. The amount of fluorescence given off by the sample was directly proportional to the concentration of tryptase in the sample. Through a radioimmunoassay method, which only detected the β form of the tryptase molecule. Eleven cases were selected as controls. These included the following cases: seven cases of sudden cardiac death and four cases of death after motor vehicle crashes.

## 3. Results

Table 1 shows the clinical history of all selected cases, the cause of the anaphylactic reaction, and the interval between the onset of symptoms and death. According to the results shown in Table 1, 72.7% of our cases did not have a history of allergy; only 1/11 had a history of asthma and celiac disease; 5/11 (45.4%) died within 1 h, and 6/11 (54.6%) within 1 min. The causes of anaphylaxis were medications (6/11), injected contrast medium (3/11), food (1/11), and latex (1/11).

The post-mortem diagnosis was based on: (1) the circumstantial evidence, including the history of exposure to a likely allergen prior to death and clinical presentation; (2) post-mortem findings suggesting an anaphylactic reaction, such as laryngeal edema, mucous plugging in the airways, erythematous skin rash and edema, eosinophilia in the mucosa and submucosa of the respiratory and the gastrointestinal tracts, and marked pulmonary congestion and edema; (3) toxicology test of serum concentrations of tryptase in femoral blood samples (from the femoral artery); (4) histological examination of all the organs using H&E; and (5) immunohistochemical examination for anti-tryptase antibody staining. The standard upper limit of total serum tryptase level has been established and was set at 40 µg/L in the Office of the Departments of Forensic Pathology.

The cases were analyzed regarding: (1) circumstantial evidence, including history of exposure; (2) post-mortem examination findings, including histological study and toxicology testing; and (3) cause of death. The data that were analyzed were extracted from the police investigation report, medical records, interviews of the victim’s family members and reports by forensic pathologists or investigators, and forensic autopsy protocols.

On the basis of these results, a new workflow as a useful tool in anaphylaxis deaths was elaborated.

### 3.1. Autopsy Findings

All cases showed pulmonary swelling and edema during autopsy. Macroscopic examination during the autopsies revealed that 64% of the cases had pharyngeal/laryngeal edema and mucus plugging in the airways. Only one case (9%) had a skin reaction that was found during the external examination. The results of toxicological analyses performed on ante- and post-mortem samples (blood and urine) were negative for alcohol, drugs and medications.

### 3.2. Histological and Immunohistochemical Analysis

**Figure 1:**
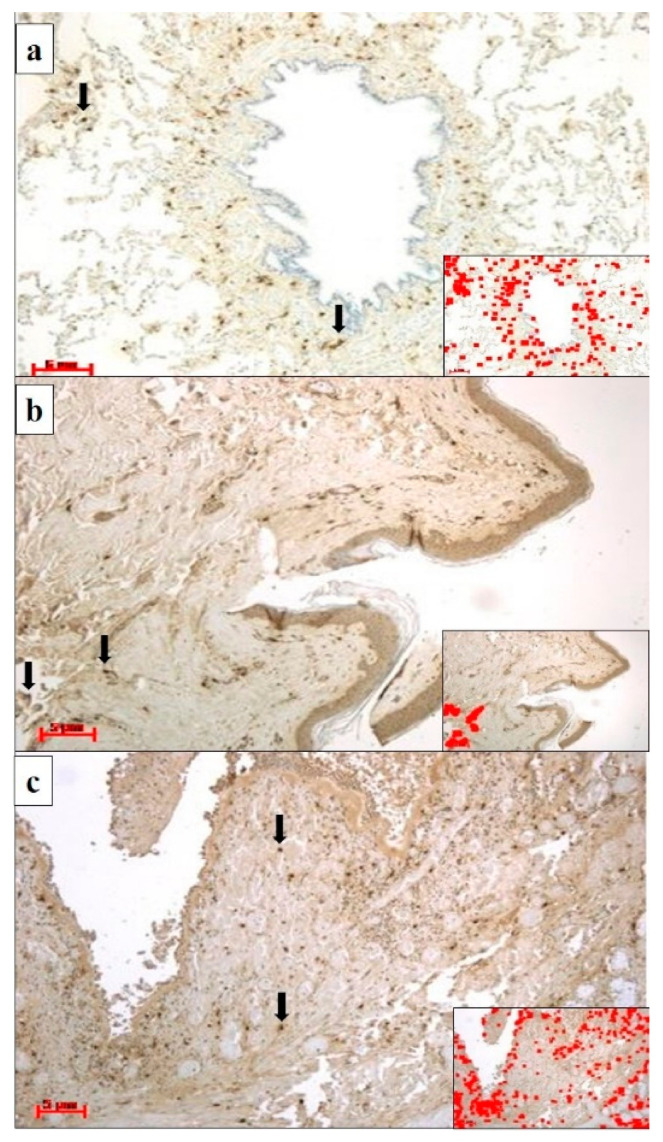
(**a**) Lung specimens from a cadaver who had died of anaphylactic shock; anti-tryptase antibody staining is strongly expressed in mast cells (black arrows) in the peribronchial interstitium. The insert shows the immunostaining software image analysis of Figure 1a, in which a highly immunostained area (red color) was detected (magnification: 20×; scale bar: 5 µm). (**b**) Skin specimens of the gluteus where medication administration occurred from a cadaver who had died of anaphylactic shock; anti-tryptase immunolocalization (black arrows) was demonstrated in the derma of the medication injection site. The insert shows the immunostaining software image analysis of Figure 1b, in which a highly immunostained area (red color) was detected (magnification: 20×; scale bar: 5 µm). (**c**) Glottis specimens of a cadaver who had died of anaphylactic shock showed strong anti-tryptase immunoexpression in mast cells (black arrows). The insert shows the immunostaining software image analysis of Figure 1c, in which a highly immunostained area (red color) was detected (magnification: 20×; scale bar: 5 µm).

**Figure 2:**
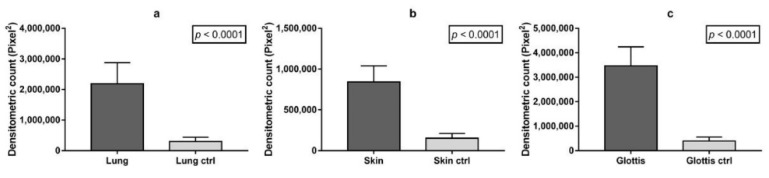
Comparison of the densitometric analysis (pixel^2^) of the tryptase immunostained area expressed by positive, dark brown pixels in the analyzed fields for: (**a**) lung tissues (*n* = 10) of cadavers who had died of anaphylactic shock vs. controls (ctrl); (**b**) skin tissue (*n* = 8) of cadavers who had died of anaphylactic shock vs. ctrl; (**c**) glottis tissue (*n* = 7) of cadavers who had died of anaphylactic shock vs. ctrl. Data are presented as mean ± standard deviation (SD) (*p* < 0.05).

**Figure 3:**
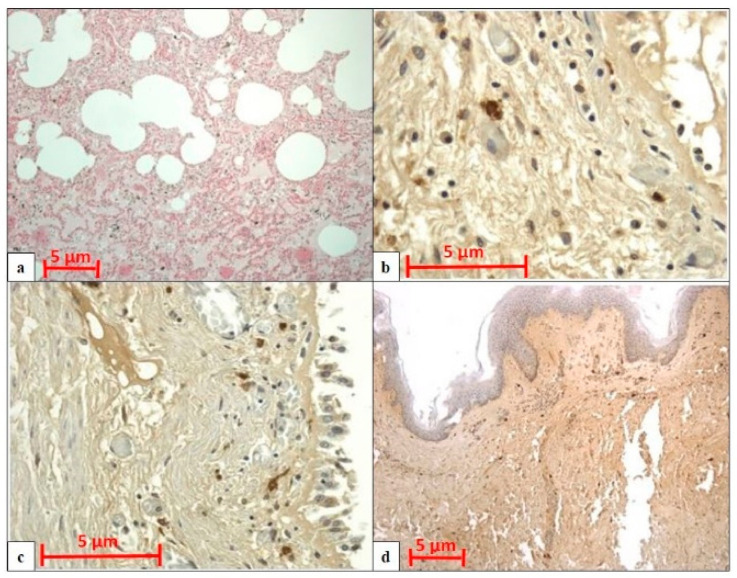
(**a**) H&E examination of lung samples shows capillary congestion and severe alveolar edema (amplification: 20×; scale bar: 5 µm). (**b**) IHC examination of lung samples with strong anti-tryptase immunopositivity (magnification: 40×; scale bar: 5 µm). (**c**) IHC examination of pharyngeal samples with strong anti-tryptase immunopositivity (magnification: 40×; scale bar: 5 µm). (**d**) IHC examination of skin samples with strong anti-tryptase immunopositivity (magnification: 20×; scale bar: 5 µm.

**Figure 4:**
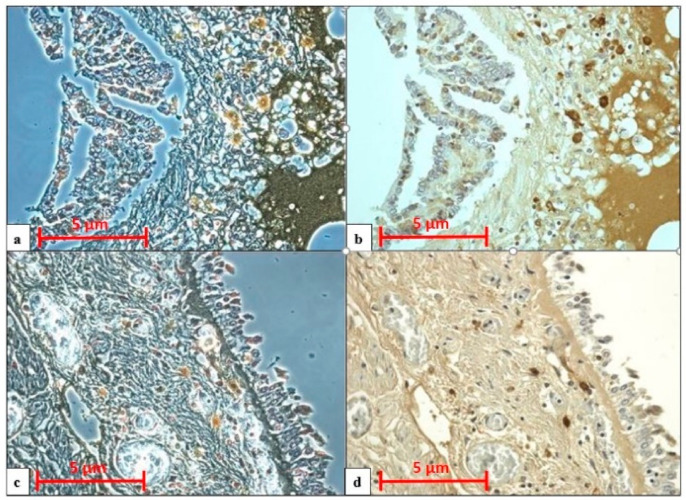
Comparison of anti-tryptase antibody reaction between confocal laser scanning microscopy (**a**,**c**) and light microscopy (**b**,**d**) shows strong anti-tryptase immunopositivity in: (**a**,**b**) lung tissue; (**c**,**d**) pharyngeal tissue. Magnification: 40×; scale bar: 5 µm.

All cases displayed pulmonary congestion and edema during the histological examination. The glottis was sampled in six cases, and the skin was sampled in eight (only in cases of transdermal administration of medication). An immunohistochemical examination of anti-tryptase antibody staining on lung samples was performed in ten autopsies, glottis in six, and skin site of injected medications in eight. All cases showed strong immunopositivity for anti-tryptase antibody staining on lung samples (10/10), on pharyngeal/laryngeal samples (7/7), and on skin samples (8/8) (Table 2). Samples from the lung, skin site of injected medications and glottis showed a strong and diffuse anti-tryptase immunolabeling. In particular, in lung specimens, anti-tryptase was found in mast cells of the connective interstitium and bronchiolar structure (Figure 1a). The skin site of medication administration also showed strong mast cell antibody immunolabelling in the connective derma (Figure 1b). Moreover, the glottis of these cadavers exhibited an overexpression of anti-tryptase antibody staining scattered in the laminar connective tissue at the vocal fold level (Figure 1c). The Shapiro–Wilk normality test showed that the level of tryptase staining intensity of positive areas (pixel^2^) in cases of anaphylactic death and controls differs significantly from a normal distribution. As shown in Figure 2, the level of tryptase staining intensity revealed that in the tissue of cadavers who had died from anaphylactic shock the tryptase immunostaining level was much higher compared to controls (*p* < 0.05). In particular, these results of tryptase immunostaining were confirmed in lung tissue (Figure 2a), skin tissue (Figure 2b), and glottis tissue (Figure 2c). Figure 3a summarizes the histological results (H&E), while in the other quadrants, the anti-tryptase immunohistochemical staining results are shown (Figure 3b–d). Figure 4 shows the anti-tryptase immunohistochemical results in lung samples by confocal laser scanning microscopy or with a light microscope (Figure 4a–d).

### 3.3. Serum Tryptase Analysis

Routine toxicology tests were performed in all eleven autopsies. Serum tryptase analysis ranged from 40.5 µg/L to 640 µg/L, and the mean value was 133.5 µg/L ± 177.9.

Table 2 shows a summary of total serum tryptase, autopsy findings, and histological examinations. All cases showed pulmonary congestion and edema during autopsy and the histological examination.

## 4. Discussion

Today, there is no specific forensic workflow in cases of death from anaphylactic shock. A systematic approach would allow forensic pathologists to arrive at a confident diagnosis of death from anaphylactic shock. Through a retrospective analysis of eleven deaths from anaphylactic shock, the aim of this study was to propose a new forensic workflow, leading to a more accurate diagnosis.

The present study shows that a typical clinical history, high levels of serum tryptase (>40 μg/L), and strong positivity for anti-tryptase antibody staining are highly suggestive for establishing the diagnosis of anaphylactic death.

Anaphylaxis is a life-threatening syndrome [3,4,5,6]. The anaphylactic reaction is mostly triggered by food and drugs, but it may be provoked by any agent capable of activating mast cells or basophils [9,24]. In the UK, about half of the 20 fatal reactions recorded each year due to anaphylactic shock are iatrogenic; the rest are caused by food ingestion or insect venom. Respiratory or cardiac arrest occurs within 30 min for food, 15 min for venom, and 5 min for iatrogenic reactions [30]. A history of exposure to anaphylactic stimuli and clinical features such as hypotension are important to identify death from anaphylactic shock [31].

Several epidemiologic and experimental studies [9,28,32,33,34,35,36,37,38,39,40,41,42,43,44,45,46] have underlined the importance of immunohistochemical analyses and the concentrations of serum tryptase; however, based on the literature, this article is the first study that combines the two parameters for a specific diagnosis of anaphylactic death.

This study showed that the symptoms of the anaphylactic reaction occurred within one hour (Table 1): one minute in the case of injected contrast medium reaction, in cases of anaphylaxis during anesthesia, the shock occurred within one minute; in cases of medication, anaphylaxis shock occurred both within one minute (60%) and within one hour (40%). These results are in agreement with previous studies [3,4,5,6].

The autopsy procedure has to be careful, with an accurate external examination, searching for injection sites of stinging or biting invertebrates, as well as blood, vitreous, and urine collection. It is essential to examine the stomach contents, above all in suspected cases of anaphylactic shock from food [47]. Autopsy findings, such as the formation of mucus plugs, congestion and intra alveolar hemorrhages, and congestion and edema of major organs, are not exhaustive or specific for the diagnosis of fatal anaphylaxis [37]. Immunohistochemical analysis using anti-tryptase antibodies is also not exhaustive for the diagnosis of death from anaphylactic shock [48,49,50].

According to Turillazzi et al. [39], in all cases, the larynx and pharynx were opened with forceps following the posterior median line and the glottis was observed; the sides were stretched outward to study the mucosa. Then, following the Ghon technique, abdominal organs were removed using the bloc method, taking care to preserve the integrity of vascular structures. All autopsies showed pulmonary congestion and edema of the lungs. When squeezing the lungs, in all cases, an abundant, reddish-colored liquid was observed. Macroscopic examination during the autopsies showed glottis edema and mucus plugging in the airways in 64% of cases. Only one case had a skin reaction that was found during the external examination.

In our study, the results of the histological and immunohistochemical analyses showed generalized stasis with areas of acute pulmonary emphysema in all autopsies, and in deaths of subjects over 40 years old, eosinophilic cross-bands ranging from segments of hypercontracted to coagulated sarcomeres in heart samples. The immunohistochemical examination of anti-tryptase antibody staining on samples from the lungs, glottis, and skin of medication injection sites revealed strong positivity for anti-tryptase antibody staining for all sampling sites in all cases. In particular, in lung specimens, anti-tryptase was found in mast cells of the connective interstitium and bronchiolar structure. Skin sites of medication administration also showed strong mast cell antibody immunolabelling in the connective derma. Moreover, the glottis of these cadavers exhibited a high level of anti-tryptase antibody staining scattered in the laminar connective tissue at the vocal fold level.

According to the guidelines on autopsy practice for suspected acute anaphylaxis of the Royal College of Pathologists [49], serum tryptase samples should always be collected, even if an autopsy is performed days or even weeks after death. Despite an average serum tryptase concentration, anaphylactic death cannot be completely excluded. Different sampling techniques can impact post-mortem tryptase levels [49,51]. A recent study demonstrated that the level of tryptase is significantly lower in samples collected via transcutaneous aspiration compared with femoral/external iliac vein samples [38]. In fact, for post-mortem tryptase analysis, a sample from a clamped femoral/external iliac vein should be defined as the gold standard [38,52,53]. There are doubts about the variability of serum tryptase by post-mortem interval (PMI). Mast cells present in the respiratory tract and heart and post-mortem cell lysis might influence the release of tryptase; for this reason, peripheral blood (i.e., femoral blood) is preferable to central blood [49,53]. After death, mast cell tryptase is very stable with a long half-life, and it could be measured up to four days after death [49].

There are four different tryptases (α, β, γ, and δ), but only the α and β form are medically necessary; during an anaphylaxis reaction, they are released by mast cells [10]. Tryptase has proinflammatory effects such as the promotion of tissue edema and remodeling, chemokine secretion, and neutrophil recruitment [10]. The tryptase level can be increased by cell autolysis or liquefaction [54]. Higher values of tryptase serum have also been found in other types of death, such as sudden infant death syndrome (SIDS), amniotic fluid embolism, and heroin-related deaths [53,54,55,56,57,58,59]. β-tryptase is a more reliable indicator of acute mast cell activation. It is emitted at the same time as histamine, but the release is slower, making this marker more suitable for post-mortem investigation [60]. In 1998, Edison et al. [51] proposed that the cut-off level of tryptase should be 10 µg/L. Subsequently, in 2007, Edston et al. [45] modified the value to over 20 (44.5 µg/L) in femoral samples. In 2011, Mayer et al. [44] recommended a cut-off level over 45 µg/L. In 2014, McLean-Tooke et al. [30] modified the cut-off level to 110 µg/L in aortic samples. Finally, in 2017, Xiao et al. [48] established a cut-off level of 43 µg/L using femoral samples. There are few studies on the change in the cut-off of tryptase levels in cases of cardiac blood samples. However, it is easy to consider that the cut-off is the same as for aortic sampling (110 µg/L) with the same reliable margin (sensitivity of 80% and specificity of 92.1%). According to Tse et al. [42], sensitivity reaches 100% when the cut-off of tryptase is between 11.4 and 30 (µg/L), but specificity is low; specificity reaches 100% when the cut-off is above 70 (µg/L). In the case of aortic blood samples, the cut-off is 110 µg/L (sensitivity 80% and specificity 92.1%) [30,37].

In this retrospective analysis, tryptase serum determination was performed as part of all autopsies (11/11). The concentration ranged from 40.5 µg/L to 640 µg/L with a median of 133.5 µg/L ± 177.9. All autopsies were performed within four days after death and all cadavers were stored at −4 °C; this did not change the validity of the test. In fact, storing a corpse at −4 °C after death does not affect tryptase levels, as has been shown by previous studies. Sravan et al. [50] performed autopsies three days after death with storage at 4 °C. Edston et al. [45] published their study in which the mean time between death and autopsy was 3.861days. Tse [42] reported two cases in which there was an analysis of tryptase levels at three days and six days after death.

The combination of anamnestic information, autopsy findings, tryptase serum determination, and immunohistochemical testing can help to make a diagnosis of anaphylactic reaction as the cause of death in patients who died suddenly with unspecific symptoms.

The post-mortem diagnosis of anaphylactic shock is a challenge, and it is often achieved by exclusion. A limitation of this study is the small sample size of the analysis. In this regard, we suggest future studies to confirm our observations.

**Figure 5:**
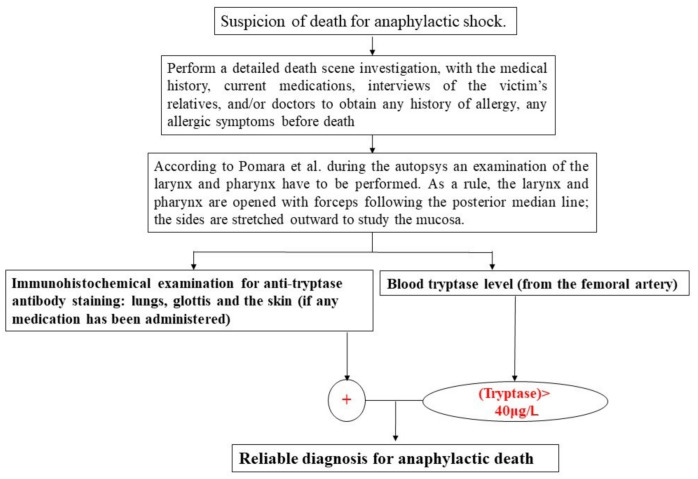
Proposed workflow to detect anaphylactic shock in fatal cases. In red text the goal of the flow chart

A sampling of serum tryptase is mandatory [49]. However, a literature review revealed that there are many doubts about its cut-off, sampling site (central or peripheral), and changes during the post-mortem interval (PMI). Histological and immunohistochemical investigation, through the use of the confocal microscope, help in the diagnosis. The results of the present study suggest that through the use of the blood tryptase concentration, together with the immunohistochemical investigation for anti-tryptase antibody staining in samples from the lung, glottis, and skin (at the site of administration of medications and contrast medium), it is possible to realize a very reliable diagnostic workflow of anaphylactic death (Figure 5). In fact, previous studies reported in the literature [61,62,63,64,65,66,67,68] have not clearly expressed how to establish a specific diagnosis of anaphylactic death. This diagnostic workflow should be used to establish an anaphylactic reaction as the cause of death with a large margin of certainty.

## Figures and Tables

**Table 1:** The circumstantial data of the selected cases.

Case Report	History of Allergy	Time to Death	Cause
Case 1	Unknown	Within 1 h	Medications
Case 2	Unknown	Within 1 min	Medications
Case 3	Drugs	Within 1 min	Medications
Case 4	Unknown	Within 1 min	Contrast medium injected
Case 5	Asthma, celiac disease	Within 1 min	Food
Case 6	Unknown	Within 1 min	Contrast medium injected
Case 7	Food	Within 1 h	Latex
Case 8	Unknown	Within 1 h	Medications
Case 9	Unknown	Within 1 h	Medications
Case 10	Unknown	Within 1 h	Medications
Case 11	Unknown	Within 1 min	Contrast medium injected

**Table 2:** Concentration of total serum tryptase, autopsy findings and histological examination. H&E: hematoxylin–eosin; IHC: immunohistochemistry.

Case Report	Tryptase Level in Blood (μg/L)	Autopsy Findings	Histological Examination
Case 1	136.5	Pulmonary congestion and edema	Pulmonary edema (H&E)
			Lung IHC not available
Case 2	130	Pulmonary congestion and edema	Pulmonary edema (H&E)
		Mucous plugging in the airways	Lung + skin from medication injection site + glottis (IHC)
		Glottis edema	
Case 3	200	Pulmonary congestion and edema	Pulmonary edema (H&E)
		Mucous plugging in the airways	Lung + skin from medication injection site (IHC)
Case 4	640	Pulmonary congestion and edema	Pulmonary edema (H&E)
		Mucous plugging in the airways	Lung (IHC)
		Glottis edema	
Case 5	41.4	Pulmonary congestion and edema	Pulmonary edema (H&E)
		Mucous plugging in the airways	Lung (IHC)
Case 6	290	Pulmonary congestion and edema	Pulmonary edema (H&E)
		Mucous plugging in the airways	Lung + skin from medication injection site (IHC)
		Glottis edema	
Case 7	133	Skin reaction	Pulmonary edema (H&E)
		Pulmonary congestion and edema	Lung + skin from medication injection site + glottis (IHC)
		Mucous plugging in the airways	
		Glottis edema	
Case 8	160	Pulmonary congestion and edema	Pulmonary edema (H&E)
		Mucous plugging in the airways	Lung + skin from medication injection site + glottis (IHC)
		Glottis edema	
Case 9	40.5	Pulmonary congestion and edema	Pulmonary edema (H&E)
		Mucous plugging in the airways	Lung + skin from medication injection site + glottis (IHC)
		Glottis edema	
Case 10	83.6	Pulmonary congestion and edema	Pulmonary edema (H&E)
		Mucous plugging in the airways	Lung + skin from medication injection site + glottis (IHC)
		Glottis edema	
Case 11	89.6	Pulmonary congestion and edema	Pulmonary edema (H&E)
		Mucous plugging in the airways	Lung + skin from medication injection site + glottis (IHC)
		Pharyngeal/laryngeal edema	

## Data Availability

All data are included in the main text.
